# Crossmodal Correspondence Mediates Crossmodal Transfer from Visual to Auditory Stimuli in Category Learning

**DOI:** 10.3390/jintelligence12090080

**Published:** 2024-08-28

**Authors:** Ying Sun, Liansheng Yao, Qiufang Fu

**Affiliations:** 1 State Key Laboratory of Brain and Cognitive Science, Institute of Psychology, Chinese Academy of Sciences, Beijing 100101, China; sunying@immu.edu.cn (Y.S.); yaols@psych.ac.cn (L.Y.); 2 University of Chinese Academy of Sciences, Beijing 101408, China; 3 College of Humanities and Education, Inner Mongolia Medical University, Hohhot 010110, China

**Keywords:** crossmodal transfer, pitch-elevation correspondence, pitch-size correspondence, category learning

## Abstract

This article investigated whether crossmodal correspondence, as a sensory translation phenomenon, can mediate crossmodal transfer from visual to auditory stimuli in category learning and whether multimodal category learning can influence the crossmodal correspondence between auditory and visual stimuli. Experiment 1 showed that the category knowledge acquired from elevation stimuli affected the categorization of pitch stimuli when there were robust crossmodal correspondence effects between elevation and size, indicating that crossmodal transfer occurred between elevation and pitch stimuli. Experiments 2 and 3 revealed that the size category knowledge could not be transferred to the categorization of pitches, but interestingly, size and pitch category learning determined the direction of the pitch-size correspondence, suggesting that the pitch-size correspondence was not stable and could be determined using multimodal category learning. Experiment 4 provided further evidence that there was no crossmodal transfer between size and pitch, due to the absence of a robust pitch-size correspondence. These results demonstrated that crossmodal transfer can occur between audio-visual stimuli with crossmodal correspondence, and multisensory category learning can change the corresponding relationship between audio-visual stimuli. These findings suggest that crossmodal transfer and crossmodal correspondence share similar abstract representations, which can be mediated by semantic content such as category labels.

## 1. Introduction

Crossmodal correspondence refers to the systematic mapping between seemingly unrelated stimuli, properties, or dimensions of different sensory modalities (Deroy and Spence 2016; Evans and Treisman 2010; Shams and Kim 2010). It has been found that crossmodal correspondence exists widely between different sensory modalities such as pitch and brightness (Evans and Treisman 2010), pitch and elevation (Jonas et al. 2017; Klapman et al. 2021), and pitch and size (Gallace and Spence 2006). Interestingly, crossmodal correspondence also exists between sensations and concepts (Campbell and Scheepers 2015; Dehaene et al. 2015) as well as actual and imaginary stimuli (Berger and Ehrsson 2018; Spence 2019). 

Although the existence of crossmodal correspondence has been widely investigated, it remains unclear what type of representation supports crossmodal correspondence between stimuli from different modalities. On the one hand, some studies suggest that crossmodal correspondence is based on modality-general or abstract representations (Erdogan et al. 2015). For example, it has been found that individuals could transfer the information received from one modality in the form of another sense of correspondence stimuli (Schmitz et al. 2021). In addition, the simultaneous presentation of crossmodal correspondence stimuli affected the individual’s judgment about unimodal objects (Amedi et al. 2001; Evans and Treisman 2010; Qi et al. 2020). On the other hand, some studies indicated that crossmodal correspondence is based on modality-specific representations (Frost et al. 2015). For example, it has been demonstrated that no multisensory brain regions play a role in crossmodal matching (Hadjikhani and Roland 1998). 

Crossmodal transfer refers to the ability that individuals can apply the knowledge acquired from one sensory modality to recognize or classify objects and stimuli in another modality (Yildirim and Jacobs 2013). It is assumed that when the acquired representation is independent of the sensory modality of perceived information, i.e., a modality-general representation, crossmodal transfer can occur (Erdogan et al. 2015; Konkle and Moore 2009; Konkle et al. 2009; Wallraven et al. 2014; Yildirim and Jacobs 2013), and when the representation is constricted in a certain connection from a perceived sensory modality, i.e., a modality-specific representation, no transfer effect can be observed (Obayashi 2004). Thus, a modified crossmodal transfer paradigm was adopted in the present study to investigate what type of representation underlies crossmodal correspondence effects. We assumed that if crossmodal transfer can occur between visual and auditory stimuli with crossmodal correspondence, it would indicate that crossmodal correspondence was based on a modality-general representation, and vice versa.

Moreover, it is crucial to investigate whether experience or learning can influence crossmodal correspondence, as this will help us understand the type of representation underlying crossmodal correspondences. On the one hand, it has been demonstrated that experiences can adjust the existing crossmodal associations and even generate new ones (Dolscheid et al. 2013; Flanagan et al. 2008; Parise 2016; Walker et al. 2010a). For example, there are significant cross-cultural differences in color-smell correspondences (Levitan et al. 2014; Nehme et al. 2016; Shankar et al. 2010) and in color-flavor associations (Spence and Velasco 2018; Velasco et al. 2014; Wan et al. 2015; Wan et al. 2016). On the other hand, it has been found that some crossmodal correspondence exists in early life (Dolscheid et al. 2014; Fernandez-Prieto et al. 2015; Pena et al. 2011), even before the acquisition of spatial relationships or abstract words (Swingley 2009). For example, 10-month-old infants tend to associate higher-frequency sounds with brighter colors and lower-frequency sounds with darker colors (Haryu and Kajikawa 2012).

Furthermore, it has been proposed that the development of pitch-elevation correspondence and pitch-size correspondence differs significantly across the human lifespan. Generally, the pitch-elevation correspondence is considered to be innate (Bien et al. 2012; Fernandez-Prieto and Navarra 2017; Korzeniowska et al. 2019; Swingley 2009), whereas the pitch-size correspondence is thought to occur relatively later (Fernandez-Prieto et al. 2015; Haryu and Kajikawa 2012). Thus, we assumed that crossmodal category learning might play different roles in pitch-elevation and pitch-size correspondence effects, and the crossmodal transfer effects might differ between pitch-elevation and pitch-size correspondences.

Therefore, the purpose of the present study was to investigate whether crossmodal transfer can occur between audio-visual stimuli with crossmodal correspondence and whether category learning mediates different types of crossmodal correspondences. To assess whether crossmodal transfer can occur, the participants were first asked to complete a crossmodal transfer session. To evaluate whether the category labels acquired in visual and auditory category learning influenced crossmodal correspondence (Chiou and Rich 2012; Evans and Treisman 2010), the category labels for visual and auditory stimuli were made congruent or incongruent with crossmodal correspondence effects in the congruent or incongruent learning condition, and the participants were asked to complete a crossmodal matching session after learning. To investigate the roles of different types of crossmodal correspondences in crossmodal transfer (Jonas et al. 2017; Pisanski et al. 2017), the pitch-elevation stimuli (Bernstein and Edelstein 1971; Walker et al. 2010b) were adopted in Experiment 1 and the pitch-size stimuli (Gallace and Spence 2006; Mondloch and Maurer 2004) in Experiments 2, 3, and 4.

## 2. Experiment 1: Adopted Pitch-Elevation Stimuli Varied in Elevation with Constant Size

Since it has been found that there is robust crossmodal correspondence between pitch and elevation stimuli, pitch-elevation stimuli were adopted in Experiment 1. The purpose of Experiment 1 was to explore whether the category knowledge acquired in elevation category learning could be transferred to the categorization of pitches and whether category learning of elevations and pitches could influence pitch-elevation and pitch-size correspondences. To assess whether crossmodal transfer could occur, the participants were asked to complete a pitch categorization task in a pre-test and a post-test before and after elevation category learning. If the accuracy for the pitch categorization task in the post-test was significantly influenced by visual category learning, it would indicate the occurrence of transfer, and vice versa. To evaluate whether elevation and pitch category learning could influence pitch-elevation correspondences, we manipulated whether the category labels they learned for pitches and elevations in category learning were either congruent or incongruent. According to previous studies (Jonas et al. 2017; Klapman et al. 2021), in the congruent condition, the labels for high elevations were the same as for high pitches and the labels for low elevations the same as for low pitches, whereas in the incongruent conditions, the labels for high elevations were the same as the labels for low pitches, and the labels for low elevations were the same as the labels for high pitches. If the selection ratio for the pitch-elevation or pitch-size matching task was significantly influenced by congruent and incongruent category learning conditions, it would indicate that the crossmodal correspondence could be mediated by the semantic labels.

### 2.1. Method

#### 2.1.1. Participants

Forty-seven right-handed and one left-handed undergraduate students (18 males and 30 females; average age: 23.9 ± 2.4) voluntarily participated and were naïve to the experiment’s purpose. They were randomly assigned to congruent and incongruent conditions. All participants reported normal hearing and normal or corrected-to-normal vision. They gave informed consent before the experiment and were paid for their attendance. The experiment, along with Experiments 2, 3, and 4, was approved by the Ethics Committee for the Protection of Participants at the Institute of Psychology, Chinese Academy of Sciences.

#### 2.1.2. Apparatus and Lab Environment

A 24-inch LED monitor with a 100 Hz refresh rate and a resolution of 1920 × 1080 pixels was utilized to present visual stimuli. Auditory stimuli were delivered through two Sony headphones, which had a response frequency of 44.1 kHz. The experiment was controlled by a desktop computer running Windows 10, using the MATLAB software package. The participants completed the experiment in a dimly lit room equipped with sound insulation, ensuring a quiet environment. To maintain a consistent viewing distance of approximately 60 cm, their heads were stabilized using a chin rest.

#### 2.1.3. Stimuli

**Figure 1:**
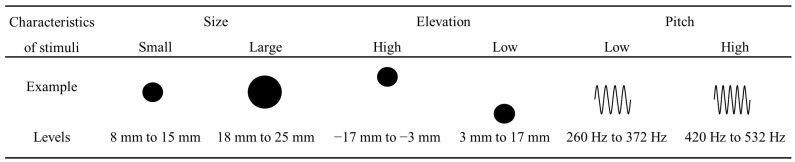
Examples of visual and auditory stimuli.

The visual stimuli were 32 black solid circles varied in elevation on a white background, which were adapted from Jonas et al.’s study (Jonas et al. 2017) (see Figure 1). The stimuli were generated by MATLAB. Specifically, the size was held constant with a radius of 16 mm, the elevation varied from 17 mm above the middle horizontal line of the screen to 17 mm below the line with a step of 2 mm. Each circle was 139 × 145 pixels, with visual angles of 2.9° and 3.1°, respectively. After excluding stimuli with a distance of −1 mm and 1 mm, eight circles with a distance from 3 mm to 17 mm were taken as “low” circles and eight circles with a distance from −17 mm to −3 mm as “high” circles. The auditory stimuli consisted of pure tones that could be described as “low” and “high” frequencies, similar to those used in the previous study (Jonas et al. 2017). A total of 16 pure tones, ranging from 260 Hz to 532 Hz in increments of 16 Hz, were used as auditory stimuli. Frequencies of 388 Hz and 404 Hz were specifically excluded from the stimulus set. That is, eight tones from 260 Hz to 372 Hz were taken as “low” pitches, while eight tones from 420 Hz to 532 Hz were taken as “high” pitches. Each pure tone was presented for a duration of 200 ms at a sound level of 50 dB, delivered through stereo headphones.

#### 2.1.4. Procedure

**Figure 2:**
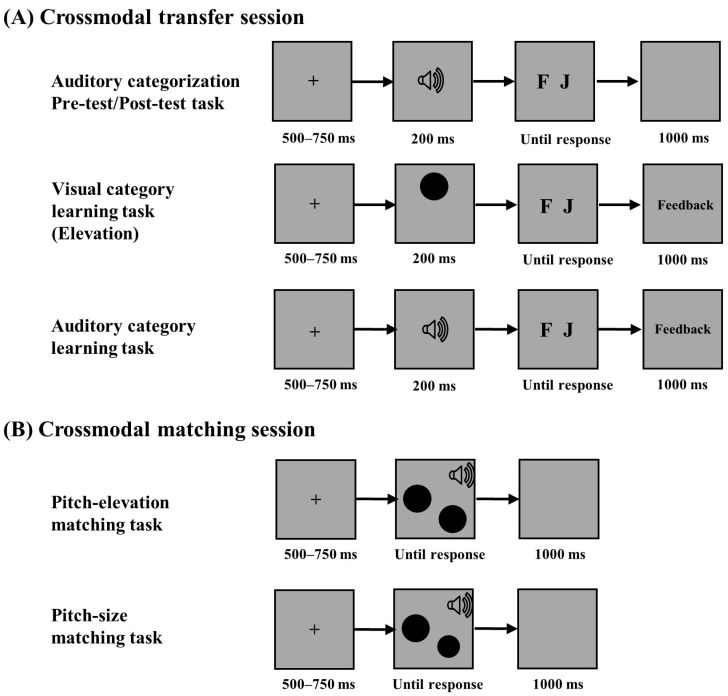
Trial procedure in Experiment 1. (**A**) Trial procedure of the auditory categorization task, the visual category learning task, and the auditory category learning task in the crossmodal transfer session. (**B**) Trial procedure of the pitch-elevation matching task and the pitch-size matching task in the crossmodal matching session.

The participants were required to engage in both a crossmodal transfer task and a crossmodal matching task. (see Figure 2). The crossmodal transfer session included two auditory categorization tasks and two category learning tasks, which were modified from the traditional two-phase transfer task. The crossmodal matching session included a pitch-elevation matching task and a pitch-size matching task.

**Crossmodal transfer session:** In the crossmodal transfer session, the participants were asked to complete an auditory categorization task in the pre-test phase and the post-test phase, a visual category learning task was inserted between the two categorization tasks, and then, there was an auditory category learning task after the post-test phase (see Figure 2A).

The participants were first asked to complete the auditory categorization task in the pre-test. In the auditory categorization task, the participants were informed that the auditory stimuli belonged to a creature either from the planet “Addie” or from the planet “Prajna” and were asked to guess which planet the sounds belonged to. Each trial commenced with a central fixation cross displayed for a duration ranging from 500 ms to 750 ms. Subsequently, a sound was presented for 200 ms, and the participants were asked to respond as quickly and accurately as possible by pressing one of two designated keys, “F” or “J”, on the keyboard. The assignment of response keys was counterbalanced across the participants. Following the response, a 1000 ms interval of a blank screen was displayed, during which no feedback was provided. There were 32 trials in the auditory categorization task.

Then, the participants were asked to complete a visual category learning task. In the visual category learning task, the trials initiated with a central fixation cross, which was displayed for 500 ms to 750 ms, followed by the presentation of a circle for 200 ms. The participants were instructed to judge whether the visual stimuli with different elevations belonged to the planet Addie or the planet Prajna as quickly and accurately as possible by pressing one of the two keys “F” and “J” on the keyboard. The response keys were counterbalanced among the participants. After the response, the word "correct" would be displayed if the judgment was correct, and the word "incorrect" would be displayed if the judgment was incorrect. The feedback was presented for 1000 ms. There were 32 trials in each block, with a short break of at least 30 s after each block. The experiment consisted of three blocks, totaling 96 trials.

After the visual category learning task, the participants were asked to complete the auditory categorization task again in the post-test. The trial procedure was the same as in the auditory categorization task in the pre-test, except that the participants were instructed to classify auditory stimuli according to the knowledge they learned in the visual category learning task.

Finally, the participants were asked to complete an auditory category learning task. The trial procedure was the same as in the visual category learning task, except that the target stimuli were auditory. To investigate whether the participants were aware of the knowledge they acquired and used in the crossmodal transfer session, they were asked to complete a self-report questionnaire after the auditory category learning task.

**Figure 3:**
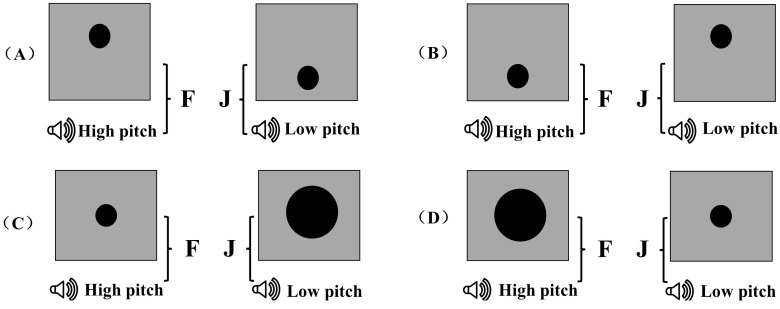
Congruent and incongruent responses in visual and auditory category learning tasks in Experiment 1. (**A**) Congruent association for pitch-elevation correspondence. (**B**) Incongruent association for pitch-elevation correspondence. (**C**) Congruent association for pitch-size correspondence. (**D**) Incongruent association for pitch-size correspondence. In the congruent condition, the category labels and responses for both auditory and visual stimuli were congruent with the established crossmodal correspondence effects. Conversely, in the incongruent condition, the labels and responses were incongruent with these effects. The response keys “F” and “J” were counterbalanced across the participants, ensuring that each key was used equally in both conditions.

**Crossmodal matching session:** In the crossmodal matching session, the participants were asked to complete a pitch-elevation matching task and a pitch-size matching task (see Figure 2B). In the pitch-elevation matching task, according to previous studies (McCormick et al. 2018), if “low” pitches were paired with “low” elevations and “high” pitches with “high” elevations, the responses were taken as congruent mappings (see Figure 3A); otherwise, the responses were taken as incongruent mappings (see Figure 3B). In the pitch-size task, according to previous studies (Bien et al. 2012), if “low” pitches were paired with “big” sizes and “high” pitches were paired with “small” sizes, the responses were taken as congruent mappings (see Figure 3C); otherwise, the responses were taken as incongruent mappings (see Figure 3D).

In the pitch-elevation matching task, each trial commenced with a central fixation cross displayed for 500 ms to 750 ms, after which a sound stimulus and a pair of circles with varying elevations were presented simultaneously. The sound was presented for only 200 ms, and the picture was presented until the response. The participants were instructed to discriminate which one of the two circles matched the sound as quickly and as accurately as possible by pressing one of the keys “F” and “J” on the keyboard. The locations of the two circles and the response keys were counterbalanced among the participants. Following each response, a 1000 ms blank screen was displayed without providing any feedback. Each block consisted of 40 trials. After each block, the participants had at least 30 s for a short rest. There were four blocks, for a total of 160 trials.

In the pitch-size matching task, the trial procedure was the same as in the pitch-elevation matching task, except that the paired circles differed in their radii. To investigate whether the participants were aware of the knowledge they used in the crossmodal matching session, they were also asked to complete a self-report questionnaire after the pitch-size matching task.

#### 2.1.5. Data Analysis

The accuracy of the auditory categorization referred to the response proportions the participants responded with the category labels that were congruent with the visual category learning task. For example, if the high elevation was labeled as being from Addie planet in category learning, the response corresponding to Addie planet for high pitches was correct. The selection ratio for the crossmodal matching session referred to the proportion of congruent and incongruent responses. Specifically, in the pitch-elevation correspondence, the congruent selection ratio referred to the proportions of the high pitch matching high elevation and low pitch matching low circles, while the incongruent selection ratio referred to the proportions of low pitch matching high circles and high pitch matching low circles. In the pitch-size matching task, the congruent selection ratio referred to the proportions of high pitch matching small circles and low pitch matching large circles, while the incongruent selection ratio referred to the proportions of high pitch matching large circles and low pitch matching small circles.

The data were analyzed using repeated-measures analysis of variance (ANOVA), complemented by Bayes factors (*BF*_10_), which were reported to quantify the evidence. The statistical software JASP, version 2022, was utilized for all computations (JASP Team, 2022). We evaluated the model evidence relative to a null model using *BF*_10_ thresholds. A *BF*_10_ value of 3 or higher indicated moderate evidence for the alternative hypothesis, while 10 or higher suggested strong evidence. Conversely, *BF*_10_ values below 0.33 indicated moderate evidence for the null hypothesis, and values below 0.1 indicated strong evidence. Values ranging from 0.33 to 3 suggested the data were equivocal, providing only weak or anecdotal evidence (Lee and Wagenmakers 2013).

### 2.2. Results

**Figure 4:**
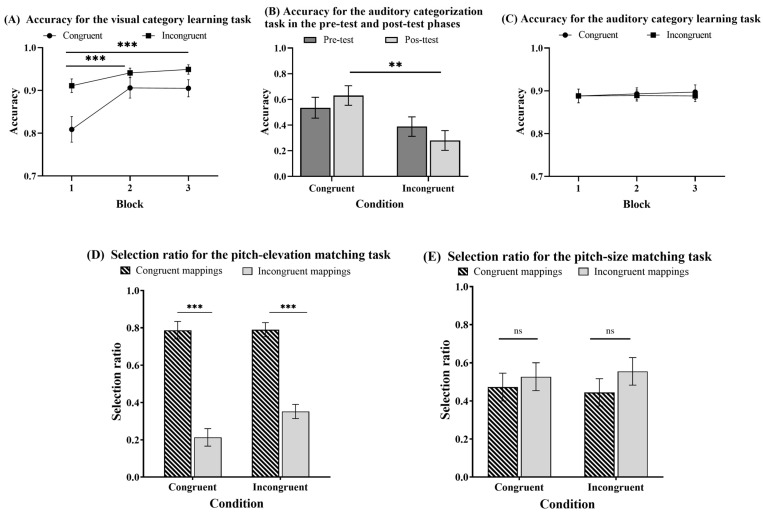
Accuracy for different tasks under congruent and incongruent conditions in Experiment 1. (**A**) Accuracy for the visual category learning task. (**B**) Accuracy for the auditory categorization task in the pre-test and post-test phases. (**C**) Accuracy for the auditory category learning task. (**D**) Selection ratio for the pitch-elevation matching task. (**E**) Selection ratio for the pitch-size matching task. Error bars represent standard errors of the mean. ns = not significant. ** *p* < .01. *** *p* < .001.

The trials with reaction times (RTs) below and above three SDs of the average RTs were excluded from the analysis, which accounted for 1.85%. Figure 4 shows the accuracies and selection ratios for different tasks under congruent and incongruent conditions in Experiment 1.

#### 2.2.1. Can People Acquire Category Knowledge in the Elevation Category Learning Task?

To evaluate whether category knowledge was acquired in elevation category learning, a 3 (block: 1–3) × 2 (condition: congruent vs. incongruent) mixed-design ANOVA was performed on the accuracy (see Figure 4A). The analysis yielded strong evidence for the significant main effect of the block factor: *F* (2, 92) = 13.009, *p* < .001, *η_p_*^2^ = 0.220, *BF*_10_ = 1261.014. A post hoc pairwise comparison with Bonferroni correction analysis revealed that the accuracy was significantly higher for the second block and the third block than the first block: *t* (46) = 4.304, *p* < .001, *d* = 0.654, *BF*_10_ = 125.646; *t* (46) = 4.523, *p* < .001, *d* = 0.687, *BF*_10_ = 109.114. There was also moderate evidence for the significant condition main effect, *F* (1, 46) = 7.381, *p* = .009, *η_p_*^2^ = 0.138, *BF*_10_ = 5.500, but anecdotal evidence for the significant block by the condition interaction, *F* (2, 92) = 3.071, *p* = .051, *η_p_*^2^ = 0.063, *BF*_10_ = 1.150. These results demonstrated that the accuracy improved with training, and the participants, for both the congruent and incongruent conditions, acquired visual category knowledge.

#### 2.2.2. Can People Transfer the Knowledge Acquired in Elevation Category Learning to the Categorization of Pitches?

To evaluate whether crossmodal transfer can occur, a 2 (experiment phase: pre-test vs. post-test) × 2 (condition: congruent vs. incongruent) mixed-design ANOVA was conducted on the accuracy (see Figure 4B). The analysis provided moderate evidence for the nonsignificant main effect of the experimental phase: *F* (1, 46) = 0.026, *p* = .873, *η_p_*^2^ = 5.602 × 10^−4^, *BF*_10_ = 0.214. However, the analysis offered moderate evidence of a significant main effect of the condition, *F* (1, 46) = 5.973, *p* = .018, *η_p_*^2^ = 0.115, *BF*_10_ = 3.175, and moderate evidence of a significant experiment phase by the condition interaction, *F* (1, 46) = 6.275, *p* = .016, *η_p_*^2^ = 0.012, *BF*_10_ = 3.495. The simple effect analysis revealed that the accuracy was significantly higher in the congruent group than in the incongruent group in the post-test phase, *t* (46) = 3.198, *p* = .013, *d* = 0.923, *BF*_10_ = 15.673, but not in the pre-test, *t* (46) = 1.343, *p* = 1.000, *d* = 0.388, *BF*_10_ = 0.589. These results indicated that the participants could transfer visual category knowledge to the categorization of auditory pitches.

#### 2.2.3. Can People Acquire Category Knowledge in the Pitch Category Learning Task?

To determine whether the participants acquired category knowledge during the pitch category learning task, a 3 (block: 1–3) × 2 (condition: congruent vs. incongruent) mixed-design ANOVA was conducted (see Figure 4C). It provided strong evidence for the nonsignificant block main effect (F (2, 92) = 0.086, *p* = .918, *η_p_*^2^ = 0.002, *BF*_10_ = 0.072), moderate evidence for the absence of a significant condition main effect (F (1, 46) = 0.070, *p* = .792, *η_p_*^2^ = 0.002, *BF*_10_ = 0.316), moderate evidence for the nonsignificant block by the condition interaction (F (2, 92) = 0.081, *p* = .922, *η_p_*^2^ = 0.002, *BF*_10_ = 0.124). However, a one-sample *t*-test was conducted and provided strong evidence that all three blocks were significantly higher than the chance level (0.05): *t* (47) = 35.393, *p* < .001, *d* = 5.108, *BF*_10_ = 1.255 × 10^32^; *t* (47) = 41.238, *p* < .001, *d* = 5.952, *BF*_10_ = 1.128 × 10^35^; *t* (47) = 36.877, *p* < .001, *d* = 5.323, *BF*_10_ = 7.777 × 10^32^. The results indicated that all participants acquired some pitch category knowledge, and there was no learning difference between the two conditions.

#### 2.2.4. Can Multimodal Category Learning Influence Matching Preference in the Pitch-Elevation Matching Task?

To assess the impact of multimodal category learning on crossmodal correspondences, a 2 (matching preference: congruent mapping vs. incongruent mapping) × 2 (condition: congruent vs. incongruent) mixed-design ANOVA was conducted on the selection ratio (see Figure 4D). The analysis provided strong evidence of a significant main effect of the matching preference, *F* (1, 46) = 91.207, *p* < .001, *η_p_*^2^ = 0.665, *BF*_10_ = 4.998 × 10^20^, indicating that the selection ratio of congruent mappings was significantly higher than incongruent mappings. However, the analysis offered moderate evidence for the absence of a significant condition main effect, *F* (1, 46) = −5.658 × 10^−14^, *p* = 1.000, *η_p_*^2^ = −1.230 × 10^−16^, *BF*_10_ = 0.233, and moderate evidence for the significant matching preference by the condition interaction, *F* (1, 46) = 0.004, *p* = .951, *η_p_*^2^ = 8.308 × 10^−5^, *BF*_10_ = 0.284. The results indicated that category learning did not influence participants’ preferences for pitch-elevation matching.

#### 2.2.5. Can Multimodal Category Learning Influence Matching Preference in the Pitch-Size Matching Task?

To evaluate whether elevation and pitch category learning can influence the pitch-size crossmodal correspondences, a 2 (matching preference: congruent mapping vs. incongruent mapping) × 2 (condition: congruent vs. incongruent) mixed-design ANOVA was performed on the selection ratio (see Figure 4E). The analysis provided moderate evidence for the nonsignificant matching preference main effect: *F* (1, 46) = 0.633, *p* = .430, *η_p_*^2^ = 0.014, *BF*_10_ = 0.365. It offered only anecdotal evidence for the nonsignificant condition main effect, *F* (1, 46) = 1.138 × 10^−14^, *p* = 1.000, *η_p_*^2^ = 2.474 × 10^−16^, *BF*_10_ = 0.234, as well as for the nonsignificant matching preference by the condition interaction, *F* (1, 46) = 0.077, *p* = .783, *η_p_*^2^ = 0.002, *BF*_10_ = 0.299. The results indicated that there were no significant pitch-size preferences, which were also not affected by elevation and pitch category learning.

### 2.3. Discussion

The results of the crossmodal transfer session showed that there was a significant difference in the accuracy of the pitch categorization task between congruent and incongruent conditions after pitch category learning in the post-test but not in the pre-test. The results indicated that the categorization of pitches can be influenced by elevation category learning, suggesting that elevation category knowledge can be transferred to the categorization of pitches. The results provided new evidence that the pitch-elevation correspondence might be based on modality-general rather than modality-specific representation. Moreover, consistent with previous findings, we found a pitch-elevation correspondence effect following visual and auditory category learning in both congruent and incongruent conditions, indicating that elevation and pitch category learning could not change the pitch-elevation correspondences.

However, unlike the pitch-elevation preference performance, no pitch-size correspondence effect was observed in both congruent and incongruent conditions. On the one hand, the results revealed that elevation and pitch category learning did not influence the corresponding mapping between size and pitch, indicating that different crossmodal correspondences might not be related to each other. On the other hand, the results were inconsistent with previous findings that showed significant pitch-size correspondences. It remains unclear whether pitch and size had crossmodal correspondence and whether category learning can influence pitch-size correspondence. To address this issue, the participants were trained with pitch and size category learning tasks under either congruent or incongruent conditions in Experiment 2.

## 3. Experiment 2: Adopted Pitch-Size Stimuli Varied in Size with Constant Elevation

It has been also suggested that the occurrence of pitch-size correspondence effects was mediated by semantic labels (Gallace and Spence 2006) or statistical coupling (Bee et al. 2000; Coward and Stevens 2004). The purpose of Experiment 2 was to explore whether category labels acquired for pitches and sizes in category learning could influence pitch-size correspondences and whether the size category knowledge could be transferred to pitch categorization. As in Experiment 1, we manipulated the category labels that were learned for pitches and sizes in the congruent and incongruent conditions separately. Specifically, in the congruent condition, the labels for small sizes were the same as for high pitches and the labels for big sizes the same as for high pitches, while in the incongruent conditions, the labels for big sizes were the same as the labels for low pitches, and the labels for small sizes were the same as the labels for high pitches. The participants were trained with the size category learning task in the crossmodal transfer session in each of the two conditions. We expected that the pitch-size correspondence effect would be influenced by pitch and size category learning and that the category knowledge acquired from sizes could be transferred to the categorization of pitches.

### 3.1. Method

#### 3.1.1. Participants

Forty-eight right-handed undergraduate students (22 males and 26 females; average age 23.4 ± 2.4) voluntarily participated and were naïve to the experiment’s purpose. They were randomly assigned to the congruent and incongruent conditions, each group with 24. All participants reported normal hearing and normal or corrected-to-normal vision. They gave informed consent before the experiment and were paid for their attendance.

#### 3.1.2. Apparatus and Lab Environment

The Apparatus and lab environment were the same as in Experiment 1.

#### 3.1.3. Stimuli

The stimuli were the same as in Experiment 1, except that the visual stimuli in visual category learning were 16 circles with different radii but at the same elevation. Specifically, the radius varied from 8 mm to 25 mm with a step of 1 mm, excluding stimuli with radii of 16 mm and 17 mm. The smallest circle was 70 × 73 pixels, with visual angles of 1.5° and 1.5°, respectively. The largest circle was 217 × 227 pixels, with visual angles of 4.8° and 4.8°, respectively. Thus, eight circles with a radius from 8 mm to 15 mm were taken as “small” circles, and eight circles with a radius from 17 mm to 25 mm were as “large” circles. The auditory stimuli were identical to those in Experiment 1.

#### 3.1.4. Procedure

The task procedures were identical to those in Experiment 1, except that the participants were trained using sizes rather than elevations in the visual category learning task, and they were asked to complete the pitch-size matching task first in the crossmodal matching session.

### 3.2. Results

**Figure 5:**
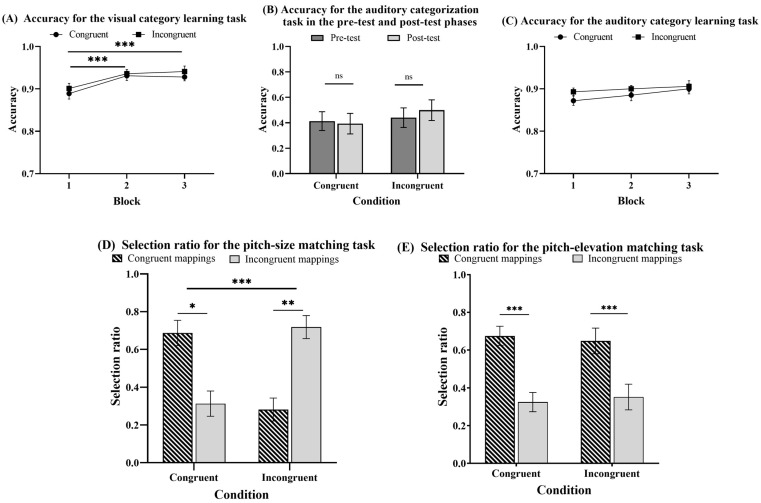
Accuracy for different tasks under congruent and incongruent conditions in Experiment 2. (**A**) Accuracy for the visual category learning task. (**B**) Accuracy for the auditory categorization task in the pre-test and post-test phases. (**C**) Accuracy for the auditory category learning task. (**D**) Selection ratio for the pitch-size matching task. (**E**) Selection ratio for the pitch-elevation matching task. Error bars represent standard errors of the mean. ns = not significant. * *p* < .05. ** *p* < .01. *** *p* < .001.

The trials with reaction times (RTs) below and above three SDs of the average RTs were excluded from the analysis, which accounted for 1.32%. Figure 5 shows the accuracies and selection ratios for different tasks under congruent and incongruent conditions in Experiment 2.

#### 3.2.1. Can People Acquire Category Knowledge in the Size Category Learning Task?

A 3 (block: 1–3) × 2 (condition: congruent vs. incongruent) mixed-design ANOVA was conducted on the accuracy (see Figure 5A). The results offered strong evidence of a significant main effect of the block: *F* (2, 92) = 9.080, *p* < .001, *η_p_*^2^ = 0.165, *BF*_10_ = 194.026. Post hoc pairwise comparisons with the Bonferroni correction showed that the accuracy significantly increased from the first to both the second and third blocks: *t* (46) = −3.627, *p* = .001, *d* = −0.678, *BF*_10_ = 25.377; *t* (46) = −3.750, *p* < .001, *d* = −0.701, *BF*_10_ = 34.110. However, the analysis provided moderate evidence for the nonsignificant condition main effect: *F* (1, 46) = 0.844, *p* = .363, *η_p_*^2^ = 0.018, *BF*_10_ = 0.305. It offered only anecdotal evidence for the nonsignificant block by the condition interaction: *F* (2, 92) = 0.078, *p* = .925, *η_p_*^2^ = 0.002, *BF*_10_ = 0.129. The results suggested that the accuracy improved with training, indicating that the participants in both conditions successfully acquired size category knowledge.

#### 3.2.2. Can People Transfer the Knowledge Acquired in Size Category Learning to the Categorization of Pitches?

A 2 (experiment phase: pre-test vs. post-test) × 2 (condition: congruent vs. incongruent) mixed-design ANOVA was conducted on the accuracy (see Figure 5B). The analysis yielded only anecdotal evidence for the nonsignificant condition main effect: *F* (1, 46) = 0.424, *p* = .518, *η_p_*^2^ = 0.009, *BF*_10_ = 0.479. It also provided moderate evidence for the nonsignificant experiment phase-main effect: *F* (1, 46) = 0.199, *p* = .657, *η_p_*^2^ = 0.004, *BF*_10_ = 0.230. Additionally, the analysis offered only anecdotal evidence for the nonsignificant interaction between the experiment phase and condition: *F* (1, 46) = 0.798, *p* = .376, *η_p_*^2^ = 0.017, *BF*_10_ = 0.379. The results indicated that the category knowledge acquired in size category learning could not influence the categorization of pitches.

#### 3.2.3. Can People Acquire Category Knowledge in the Pitch Category Learning Task?

A 3 (block: 1–3) × 2 (condition: congruent vs. incongruent) mixed-design ANOVA was conducted on the accuracy (see Figure 5C). The analysis provided only anecdotal evidence for the nonsignificant block main effect, *F* (2, 92) = 2.002, *p* = .141, *η_p_*^2^ = 0.042, *BF*_10_ = 0.404, and for the nonsignificant condition main effect, *F* (1, 46) = 1.714, *p* = .197, *η_p_*^2^ = 0.036, *BF*_10_ = 0.460. Additionally, it offered moderate evidence for the nonsignificant block by the condition interaction: *F* (2,92) = 0.253, *p* = .777, *η_p_*^2^ = 0.005, *BF*_10_ = 0.146. However, a one-sample *t*-test was conducted and provided strong evidence that all three blocks were significantly higher than the chance level (0.05): *t* (47) = 53.791, *p* < .001, *d* = 7.764, *BF*_10_ = 1.770 × 10^40^; *t* (47) = 52.371, *p* < .001, *d* = 7.559, *BF*_10_ = 5.272 × 10^39^; *t* (47) = 45.343, *p* < .001, *d* = 6.545, *BF*_10_ = 7.923 × 10^36^. The results indicated that participants in both conditions expressed some category knowledge, and there was no difference between the two conditions.

#### 3.2.4. Can Multimodal Category Learning Influence Matching Preferences in the Pitch-Size Matching Task?

A 2 (matching preference: congruent mappings vs. incongruent mappings) × 2 (condition: congruent vs. incongruent) mixed-design ANOVA was conducted on the selection ratio (see Figure 5D). The analysis provided moderate evidence for the nonsignificant matching preference main effect: *F* (1, 46) = 0.683, *p* = .413, *η_p_*^2^ = 0.015, *BF*_10_ = 0.467. There was anecdotal evidence for the nonsignificant main effect of the condition: *F* (1, 46) = −6.894 × 10^−14^, *p* = 1.000, *η_p_*^2^ = −1.499 × 10^−15^, *BF*_10_ = 0.231. However, there was strong evidence of a significant matching preference by the condition interaction: *F* (1, 46) = 19.701, *p* < .001, *η_p_*^2^ = 0.300, *BF*_10_ = 5.671. The simple effect analysis revealed that the selection ratio of matching preference was significantly higher for congruent than incongruent in the congruent condition (*t* (46) = 2.772, *p* = .011, *d* = 0.566, *BF*_10_ = 4.524) but significantly lower for incongruent than congruent in the incongruent condition (*t* (46) = −3.547, *p* = .002, *d* = −0.724, *BF*_10_ = 21.871). The results indicated that category learning influenced the matching preference in the pitch-size matching task.

#### 3.2.5. Can Multimodal Category Learning Influence Matching Preference in the Pitch-Elevation Matching Task?

A 2 (matching preference: congruent mapping vs. incongruent mapping) × 2 (condition: congruent vs. incongruent) mixed-design ANOVA was performed on the selection ratio (see Figure 5E). There was strong evidence of a significant main effect for the matching preference: *F* (1, 46) = 63.728, *p* < .001, *η_p_*^2^ = 0.710, *BF*_10_ = 9.634 × 10^12^. However, there was only anecdotal evidence for the nonsignificant main effect of the condition, *F* (1, 46) = −4.546 × 10^−14^, *p* = 1.000, *η_p_*^2^ = −11.749 × 10^−15^, *BF*_10_ = 0.299, as well as for the nonsignificant interaction between the matching preference and condition, with *F* (1, 46) = 0.053, *p* = .819, *η_p_*^2^ = 0.002, *BF*_10_ = 0.415. These results indicated a robust pitch-elevation correspondence effect, suggesting that visual and auditory category learning did not significantly influence the mapping preferences in pitch-elevation matching.

### 3.3. Discussion

Unlike in Experiment 1, the results revealed that there was no significant difference in the accuracy for the pitch categorization task between congruent and incongruent learning conditions in the pre-test and post-test phases. The results indicated that the category knowledge acquired in size category learning might not be transferred to the categorization of pitches. Moreover, the congruent and incongruent learning conditions influenced the selection ratio for the pitch-size matching task. Specifically, in the congruent learning condition, the selection ratio for congruent mappings was significantly higher than for incongruent mappings. Conversely, in the incongruent learning condition, the selection ratio for congruent mappings was significantly lower than for incongruent mappings. The results suggested that the pitch-size matching preference was determined by the category labels that were learned in category learning, providing new evidence for the semantic mediation of crossmodal mappings.

Moreover, a robust pitch-elevation correspondence effect was observed in the pitch-elevation task, and the size and pitch category learning tasks did not influence the preference in the pitch-elevation task. On the one hand, the results confirmed that a change in pitch-size correspondence did not influence pitch-elevation matching, indicating that different crossmodal correspondences might be independent of each other. On the other hand, the results suggested that the pitch-size correspondence might not be as robust as the pitch-elevation correspondence, which might be the reason for no crossmodal transfer between sizes and pitches in Experiment 2.

## 4. Experiment 3: Adopted Pitch-Size Stimuli Varied in Size with Constant Elevation

In Experiment 1, the participants may have utilized the fixation cross as a reference for categorizing elevations (Gallace and Spence 2006), but there was no such reference for the size judgments in Experiment 2. This discrepancy could potentially account for the different results observed in Experiments 1 and 2. To test this possibility, we added a gray square around the cross, which can be taken as a reference in the size judgment in Experiment 3. The manipulation of congruent and incongruent conditions was identical to Experiment 2. We expected that the pitch-size correspondence effect would be influenced by pitch and size category learning and that the category knowledge acquired from sizes can be transferred to the categorization of pitches.

### 4.1. Method

#### 4.1.1. Participants

Forty-eight right-handed undergraduate students (21 males and 27 females; average age 19.85 ± 1.09) voluntarily participated and were naïve to the experiment’s purpose. They were randomly assigned to the congruent and incongruent conditions, each group with 24. All participants reported normal hearing and normal or corrected-to-normal vision. They gave informed consent before the experiment and were paid for their attendance.

#### 4.1.2. Apparatus and Lab Environment

The Apparatus and lab environment were the same as in Experiment 2.

#### 4.1.3. Stimuli

The stimuli were identical to those in Experiment 2, except that a gray square around the cross was added in the visual category learning task.

#### 4.1.4. Procedure

**Figure 6:**
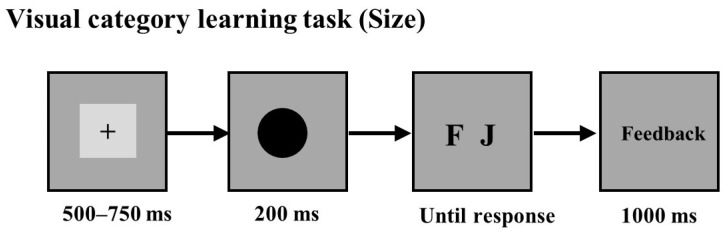
Trial procedure of the visual category learning task in Experiment 3.

In Experiment 3, the procedure was the same as in Experiment 2, except that a gray square with a diameter of 16 mm and an RGB value of [198,198,198] was simultaneously presented in the background of the cross-fixation point in the visual category task and the pitch-size mapping task (see Figure 6).

### 4.2. Results

**Figure 7:**
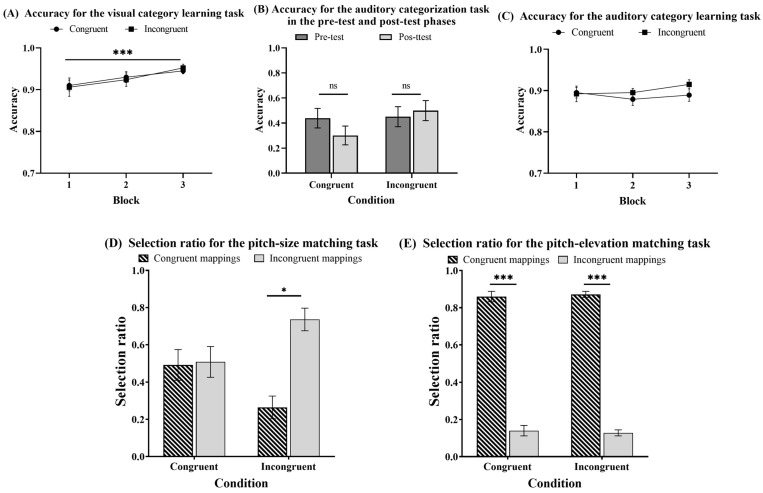
Accuracy for different tasks under congruent and incongruent conditions in Experiment 3. (**A**) Accuracy for the visual category learning task. (**B**) Accuracy for the auditory categorization task in the pre-test and post-test phases. (**C**) Accuracy for the auditory category learning task. (**D**) Selection ratio for the pitch-size matching task. (**E**) Selection ratio for the pitch-elevation matching task. Error bars represent standard errors of the mean. ns = not significant. * *p* < .05. *** *p* < .001.

The trials with reaction times (RTs) below and above three SDs of the average RTs were excluded from the analysis, which accounted for 2.00%. Figure 7 shows the accuracies and selection ratios for different tasks under congruent and incongruent conditions in Experiment 3.

#### 4.2.1. Can People Acquire Category Knowledge in the Size Category Learning Task?

A 3 (block: 1–3) × 2 (condition: congruent vs. incongruent) mixed-design ANOVA was performed on the accuracy (see Figure 7A). This analysis provided strong evidence of a significant block main effect: *F* (2, 92) = 8.297, *p* < .001, *η_p_*^2^ = 0.153, *BF*_10_ = 67.545. Post hoc pairwise comparisons with the Bonferroni correction revealed a significantly higher accuracy for the third block compared to the first block: *t* (46) = −4.056, *p* < .001, *d* = −0.646, *BF*_10_ = 55.073. However, there was moderate evidence for the nonsignificant main effect of the condition, with *F* (1, 46) = 0.064, *p* = .802, *η_p_*^2^ = 0.001, *BF*_10_ = 0.285, as well as for the nonsignificant block by the condition interaction, *F* (2, 92) = 0.007, *p* = .993, *η_p_*^2^ = 1.502 × 10^−4^, *BF*_10_ = 0.188. These results indicated that the accuracy increased with training, suggesting that the participants in both conditions learned size category knowledge.

#### 4.2.2. Can People Transfer the Knowledge Acquired in Size Category Learning to the Categorization of Pitches?

A 2 (experiment phase: pre-test vs. post-test) × 2 (condition: congruent vs. incongruent) mixed-design ANOVA was conducted on the accuracy (see Figure 7B). The analysis offered only anecdotal evidence for the nonsignificant main effect of the experiment phase: *F* (1, 46) = 0.634, *p* = .430, *η_p_*^2^ = 0.014, *BF*_10_ = 0.273. It provided moderate evidence for the nonsignificant main effect of the condition: *F* (1, 46) = 1.210, *p* = .277, *η_p_*^2^ = 0.026, *BF*_10_ = 0.513. There was moderate evidence for the nonsignificant interaction between the experiment phase and the condition: *F* (1, 46) = 2.724, *p* = .106, *η_p_*^2^ = 0.026, *BF*_10_ = 0.804. The results indicated that the category knowledge acquired in size category learning could not influence the categorization of pitches.

#### 4.2.3. Can People Acquire Category Knowledge in the Pitch Category Learning Task?

A 3 (block: 1–3) × 2 (condition: congruent vs. incongruent) mixed-design ANOVA was conducted on the accuracy (see Figure 7C). The analysis provided only anecdotal evidence for the nonsignificant experiment-phase main effect, *F* (2, 92) = 1.132, *p* = .327, *η_p_*^2^ = 0.024, *BF*_10_ = 0.394, and for the nonsignificant condition main effect, *F* (1, 46) = 0.661, *p* = .420, *η_p_*^2^ = 0.014, *BF*_10_ = 0.177. Additionally, it offered moderate evidence for the nonsignificant experiment phase by the condition interaction: *F* (2, 92) = 0.966, *p* = .385, *η_p_*^2^ = 0.021, *BF*_10_ = 0.261. However, a one-sample *t*-test was conducted and provided strong evidence that all three blocks were significantly higher than the chance level (0.05), *t* (47) = 34.526, *p* < .001, *d* = 4.983, *BF*_10_ = 4.184 × 10^31^; *t* (47) = 42.282, *p* < .001, *d* = 6.103, *BF*_10_ = 3.450 × 10^35^; *t* (47) = 43.323, *p* < .001, *d* = 6.253, *BF*_10_ = 1.025 × 10^36^. The results indicated that participants acquired some auditory category knowledge in both conditions, and there was no difference between the two conditions.

#### 4.2.4. Can Multimodal Category Learning Influence Matching Preferences in the Pitch-Size Matching Task?

A 2 (matching preference: congruent mappings vs. incongruent mappings) × 2 (condition: congruent vs. incongruent) mixed-design ANOVA was conducted on the selection ratio (see Figure 7D). The analysis yielded strong evidence of a significant main effect of matching preference, *F* (1, 46) = 5.639, *p* = .022, *η_p_*^2^ = 0.109, *BF*_10_ = 18.421, indicating that the matching preference substantially influenced the preferences. However, there was only moderate evidence for the nonsignificant main effect of condition: *F* (1, 46) = 0.314, *p* = .755, *d* = 0.000, *BF*_10_ = 0.215. Importantly, there was strong evidence of a significant interaction between the matching preference and condition: *F* (1, 46) = 4.883, *p* = .032, *η_p_*^2^ = 0.096, *BF*_10_ = 14.807. Simple effect analysis revealed that the selection ratio of congruent mapping was significantly lower than that of incongruent mapping in the incongruent condition, *t* (46) = 2.375, *p* = .022, *d* = 0.686, *BF*_10_ = 1.644, while there was no significant difference between the selection ratio of congruent trials and incongruent mapping in the congruent condition, *t* (46) = −0.117, *p* = 1.00, *d* = −0.048, *BF*_10_ = 0.216. The results indicated that category learning influenced participants’ preferences for pitch-size matching in the incongruent condition rather than the congruent condition.

#### 4.2.5. Can Multimodal Category Learning Influence Matching Preferences in the Pitch-Elevation Matching Task?

A 2 (matching preference: congruent mappings vs. incongruent mappings) × 2 (conditions: congruent vs. incongruent) mixed-design ANOVA was conducted on the selected ratio (see Figure 7E). The analysis provided strong evidence for the significant main effect of the matching preference: *F* (1, 46) = 91.207, *p* < .001, *η_p_*^2^ = 0.665, *BF*_10_ = 4.919 × 10^20^. However, it provided moderate evidence for the nonsignificant condition main effect: *F* (1, 46) = −5.658 × 10^−14^, *p* = 1.000, *η_p_*^2^ = −1.230 × 10^−16^, *BF*_10_ = 0.233. Additionally, there was only anecdotal evidence for the nonsignificant interaction between the matching preference and condition, *F* (1, 46) = 0.004, *p* = .951, *η_p_*^2^ = 8.308 × 10^−5^, *BF*_10_ = 0.284. These results indicated a robust pitch-elevation correspondence effect that appeared to be unaffected by size and pitch category learning.

### 4.3. Discussion

As in Experiment 2, no crossmodal transfer effect was observed between size and pitch even though a reference for the size judgment was added, confirming that the size category knowledge could not be transferred to the categorization of pitches. Importantly, the results of the pitch-elevation matching task principally replicated the results in Experiment 2, indicating that the direction of pitch-size mapping was modulated by the labels the participants acquired in visual and auditory category learning. Thus, we assumed that there being no crossmodal transfer effect between sizes and pitches might have been due to the absence of a robust crossmodal correspondence effect between pitch and size. To examine this possibility, Experiment 4 was conducted.

## 5. Experiment 4: Adopted Pitch-Size Stimuli Varied in Size with Constant Elevation

The purpose of Experiment 4 was to further investigate whether there was robust crossmodal correspondence between pitch and size and whether only size category learning can affect pitch-size mapping. To assess the issues, a pitch-size matching task was first conducted, and then, a size category learning task and a pitch-size matching task were conducted. Based on the results in Experiments 2 and 3, we expected that there would be no robust pitch-size correspondence effect and that the effect would not be influenced by size category learning.

### 5.1. Method

#### 5.1.1. Participants

Twenty-eight right-handed undergraduate students (9 males and 19 females; average age 22.3 ± 2.3) voluntarily participated in this experiment and were naïve to the experiment’s purpose. All participants reported normal hearing and normal or corrected-to-normal vision. They gave informed consent before the experiment and were paid for their attendance.

#### 5.1.2. Apparatus and Lab Environment

The Apparatus and lab environment were the same as in Experiment 3.

#### 5.1.3. Stimuli

In the visual category learning task, the sizes were identical to those in Experiment 3. In the pitch-size matching task, the auditory and visual stimuli were the same as in Experiment 3.

#### 5.1.4. Procedure

The participants were asked to complete three tasks: a pitch-size matching task, a size category learning task, and a pitch-size matching task. The procedure of each task was the same as in Experiment 3.

### 5.2. Results

**Figure 8:**
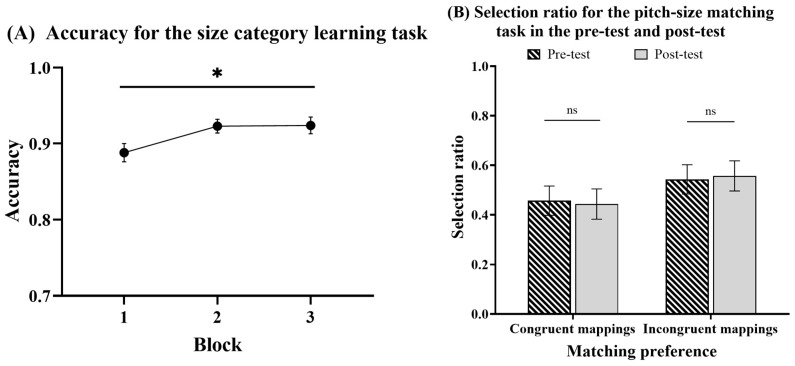
Accuracy for size category learning task and selection ratio for pitch-size matching task in Experiment 4. (**A**) Accuracy for size category learning task. (**B**) Selection ratio for the pitch-size matching task in the pre-test and post-test. Error bars represent standard errors of the mean. ns = not significant. * *p* < .05.

The trials with reaction times (RTs) below and above three SDs of the average RTs were excluded from the analysis, which accounted for 1.68%. Figure 8 shows the accuracies and selection ratios for different tasks under congruent and incongruent conditions in Experiment 4.

#### 5.2.1. Can People Acquire Category Knowledge in the Size Category Learning Task?

A one-way repeated ANOVA with a block (1–3) was conducted (see Figure 8A). The analysis provided moderate evidence of a significant main effect of the block, *F* (2, 54) = 4.048, *p* = .023, *η_p_*^2^ = 0.130, *BF*_10_ = 2.929. Post hoc pairwise comparisons with the Bonferroni correction showed that the accuracy significantly increased from the first to both the second block (*t* (27) = −2.42, *p* = .046, *d* = −0.458, *BF*_10_ = 6.248) and the third block (*t* (27) = −2.502, *p* = .046, *d* = −0.473, *BF*_10_ = 1.203). These results suggested that the participants acquired size category knowledge.

#### 5.2.2. Can Size Category Learning Influence Matching Preference in the Pitch-Size Matching Task?

A 2 (matching preference: congruent mappings vs. incongruent mappings) × 2 (experiment phase: pre-test vs. post-test) repeated ANOVA was conducted on the selection ratio (see Figure 8B). The analysis provided only anecdotal evidence for the nonsignificant main effect of the matching preference: *F* (1, 27) = 0.729, *p* = .407, *η_p_*^2^ = 0.026, *BF*_10_ = 0.697. Additionally, there was moderate evidence for the nonsignificant main effect of the experiment phase (*F* (1, 27) = 1.888 × 10^−13^, *p* = 1.000, *η_p_*^2^ = 6.994 × 10^−15^, *BF*_10_ = 0.196) and for the nonsignificant interaction between the matching preference and experiment phase (*F* (1, 27) = 0.340, *p* = .565, *η_p_*^2^ = 0.012, *BF*_10_ = 0.278). The results indicated that there was no significant crossmodal correspondence between pitch and size, and only visual category learning did not influence the participants’ preferences for pitch-size matching.

### 5.3. Discussion

The results revealed that there was no significant matching preference in the pitch-size mapping tasks in both the pre-test and post-test, indicating no significant pitch-size correspondence effect, and only size category learning did not influence the pitch-size mapping preference. This is consistent with previous findings that a higher-frequency tone did not always associate with a smaller object and a lower-frequency tone with a larger object (Haryu and Kajikawa 2012; Pisanski et al. 2014).

## 6. General Discussion

The results revealed that elevation category knowledge can be transferred to the categorization of pitches when there was a robust correspondence effect between elevation and pitch, but size category knowledge cannot be transferred to the categorization of pitches when there was no robust correspondence effect between size and pitch. The results indicated that crossmodal correspondence could mediate the occurrence of crossmodal transfer, and crossmodal correspondence was based on modality-general knowledge. Moreover, visual and auditory category learning can influence the preferences in the pitch-size matching mask but not in the pitch-elevation matching task. The results suggest that visual and auditory category learning can affect and promote the formation of crossmodal correspondence when there is no robust crossmodal correspondence between audio-visual stimuli, and crossmodal correspondence can be mediated by semantic content such as category labels.

### 6.1. Crossmodal Correspondence Can Mediate the Occurrence of Crossmodal Transfer

The results of Experiment 1 revealed that visual category learning of elevations can be transferred to the auditory categorization of pitches when there were robust crossmodal correspondence effects between elevations and pitches. Consistently, previous studies have found that crossmodal correspondence can facilitate crossmodal transfer and have a top-down effect on it (Shapiro et al. 2009). For example, when there is a correspondence between visual and auditory information, people can spontaneously create symbols for communication, allowing information to be transferred between different sensory modalities (Schmitz et al. 2021). Nevertheless, previous studies used only two extreme stimuli, while the current study adopted sixteen continuous stimuli, as mapping stimuli need to be used as stimuli in category learning tasks. Our findings provided new evidence that crossmodal transfer can occur between visual and auditory stimuli with crossmodal correspondence, and the crossmodal correspondence, at least for the pitch-elevation correspondence, is based on modality-general representations.

However, the results of Experiments 2 and 3 showed that the size category knowledge cannot be transferred to the categorization of pitches even though the pitch-size correspondence effect was influenced by visual and auditory category learning. The results of Experiment 4 confirmed that no crossmodal transfer between sizes and pitches might be due to no robust pitch-size correspondence. There might be two reasons for nonsignificant pitch-size correspondence effects in the current study. Firstly, previous studies showing a significant pitch-size correspondence effect often used only two sizes and two pitches, while the current study used eight continuous sizes and eight pitches. The continuous stimuli used in the current study might have made it difficult for the participants to label each of them and, thus, despair the association between pitches and sizes. Consistently, previous findings suggested that the pitch-size correspondence was mediated by semantic labels and was relative in nature (Brunetti et al. 2018), and our results in Experiments 2 and 3 also indicated that the pitch-size mappings could be shaped by pitch and size category learning. Secondly, previous studies recruited Western people, while this study recruited Chinese people. There might be a cross-culture difference in the pitch-size correspondence, as previous studies have demonstrated that there were cross-culture differences in the categorization of visual stimuli between Western and Eastern people (Šašinková et al. 2023).

Crossmodal transfer can occur when there were robust crossmodal correspondence effects but cannot occur when there were no crossmodal correspondence effects. Our results suggest that crossmodal correspondence can be taken as an important factor that mediates the occurrence of crossmodal transfer. Previous studies have found that crossmodal transfer cannot occur when information from multiple senses does not initially match in format (Schumacher et al. 2016) or when the two sensory modalities do not interact during the previous processing (Harrap et al. 2019). We extended them by demonstrating that crossmodal transfer cannot occur when the crossmodal stimuli do not have robust crossmodal correspondence, which can be mediated by semantic factors such as category labels.

### 6.2. Multimodal Category Learning Can Shape Crossmodal Correspondence between Visual and Auditory Stimuli

The results of Experiments 2 and 3 revealed that when participants learned congruent category labels, they tended to respond with congruent preferences in the matching task, but when they learned incongruent category labels, they tended to respond with incongruent preferences in the mapping task. Our results indicated that semantic labels can mediate the formation of crossmodal correspondences, which is consistent with previous findings (Dolscheid et al. 2013; Fernandez-Prieto and Navarra 2017; Fernandez-Prieto et al. 2015; Shen and Porat 2017). For example, it has been found that visual and auditory category learning can shape the formation of pitch-size correspondence (Fernandez-Prieto and Navarra 2017; Fernandez-Prieto et al. 2017; Shen and Porat 2017), and it was suggested that statistical structure is not the only reason for pitch-size correspondence (Ernst 2007; Spence 2011, 2022). Considering pitch-size correspondence was more pronounced in 6-month-old infants than in 4-month-olds (Fernandez-Prieto et al. 2015), our results indicated that the pitch-size correspondence might be mainly mediated by semantic factors such as category labels.

Moreover, although the opposite preference patterns were observed in the pitch-size matching task between congruent and incongruent conditions, the mapping preference was still consistent with classical correspondence effects in the pitch-elevation matching task in Experiments 2 and 3. That is, in most trials, high-pitch sounds matched with high-elevation circles and low-pitch sounds with low-elevation circles. These results suggested that a change in the pitch-size correspondence would not affect the mapping preferences in the pitch-elevation matching task, suggesting that crossmodal correspondence may be a multiple-mapping relationship rather than a single mechanism.

Furthermore, there was a robust pitch-elevation correspondence effect regardless of the learning condition being congruent or incongruent, indicating that multimodal category learning did not influence the mapping between pitch-elevation correspondence in the current study. In fact, in a pilot study we are currently conducting, we found that even after seven consecutive days of crossmodal category learning, the direction of the pitch-elevation correspondence could not be reversed. This was inconsistent with previous findings, which found that sufficient training in the reversal relationship between size and weight could lead to system changes in judging the negative load when lifting (Flanagan et al. 2008). However, this was consistent with previous findings that pitch-elevation correspondence has been observed in three- to four-month-old preverbal infants (Walker et al. 2010b). These finding suggest that pitch-elevation correspondence may be inherently arbitrary in the brain.

### 6.3. Limitations

There were some limitations in the current study. Firstly, only two pairs of crossmodal correspondence stimuli from visual and auditory modalities were selected as experimental materials in this study; more types of crossmodal correspondence stimuli are needed to investigate the generalizability of this conclusion. Secondly, consistent with previous studies, there was a robust pitch-elevation correspondence effect, but inconsistent with previous studies, no significant pitch-size correspondence effect was observed in this study. Future studies need to further examine what factors can influence the occurrence of pitch-size correspondence and whether crossmodal transfer can occur between sizes and pitch if a significant pitch-size correspondence effect is found. Thirdly, this study mainly focused on the transfer of category knowledge from the visual to the auditory modality. Future studies need to further investigate whether crossmodal transfer is bidirectional.

## 7. Conclusions

To conclude, our findings provided new evidence that crossmodal correspondence can mediate the occurrence of crossmodal transfer and that crossmodal correspondence can be based on modality-general representation. Interestingly, we also found that multimodal category learning can affect and promote the formation of crossmodal correspondence when there is no robust crossmodal correspondence between audio-visual stimuli, suggesting that crossmodal correspondence can be mediated by semantic factors such as category labels. These findings suggest that crossmodal transfer and crossmodal correspondence share similar abstract representations, which are mediated by semantic content such as category labels.

## Data Availability

Due to participant privacy concerns, the experimental data are currently not publicly available. If there is a legitimate need, please contact the author to obtain the data.
